# Inflammatory Markers and MicroRNAs: The Backstage Actors Influencing Prognosis in Colorectal Cancer Patients

**DOI:** 10.3390/ijms19071867

**Published:** 2018-06-26

**Authors:** Rihab Nasr, Miza Salim Hammoud, Farah Nassar, Deborah Mukherji, Ali Shamseddine, Sally Temraz

**Affiliations:** Department of Internal Medicine, American University of Beirut Medical Center, Riad El Solh, Beirut 110 72020, Lebanon; rn03@aub.edu.lb (R.N.); ms302@aub.edu.lb (M.S.H.); fn17@aub.edu.lb (F.N.); dm25@aub.edu.lb (D.M.); as04@aub.edu.lb (A.S.)

**Keywords:** colorectal cancer, microRNA, inflammatory markers, prognosis, survival outcomes

## Abstract

Background: Colorectal cancer (CRC) remains a deadly disease, afflicting the lives of millions worldwide. The prognosis of CRC patients is best predicted by surgical resection and pathological analysis of specimens. Emerging evidence has attributed a significant role to inflammatory markers and microRNAs (miRNAs) in the prognosis and survival of CRC patients. Aim: Here, we review the literature on inflammatory markers and miRNAs with an established role on survival rates, response to systemic chemotherapy, and other clinic-pathological parameters in CRC patients. Results: Our literature review revealed a critical role of inflammatory markers—specifically, the acute-phase proteins, inflammatory cytokines, and blood cell ratios—on prognostic outcomes in CRC patients. MiRNAs, on the other hand, were useful in predicting prognosis and clinical response and accordingly stratifying CRC patients for optimal drug selection. Conclusion: These biomarkers are easily measured in routine blood exams and can be used in adjunct to the tumor-node-metastasis (TNM) staging system to identify high-risk patients and those who are more likely to benefit from chemotherapy and other targeted therapies. However, more prospective studies are needed for the validation of these discussed prognostic and predictive biomarkers.

## 1. Introduction

Worldwide, colorectal cancer (CRC) remains one of the main causes of cancer death, with an incidence approaching over 1.4 million annually [1]. Survival among patients with stage II CRC approaches 70–80%; however, around 30% will relapse. Still, there is no reliable biomarker to determine which stage II patients are at high risk and which should be managed with adjuvant chemotherapy. As for advanced stages III and IV, there is also a lack of reliable prognostic biomarkers that determine which patients will benefit from chemotherapy. In both early and advanced stages of the disease, identification of biomarkers that would improve CRC prognostication is required for better clinical management.

There is growing evidence that inflammation is involved in tumor etiology and that the ongoing systemic inflammatory response is associated with worse prognosis in numerous cancers [2]. Consequently, many studies have investigated the predictive and prognostic role of various inflammatory markers, such as c-reactive protein (CRP), albumin, haptoglobin, d-dimer, ferritin, fibrinogen, neutrophil–lymphocyte ratio, lymphocyte–monocyte ratio, and platelet–lymphocyte ratio [3,4,5,6,7,8], on response and survival outcomes in the setting of CRC.

MicroRNAs (miRNAs) are small, noncoding, single-stranded RNA molecules with crucial regulatory functions, including proliferation, apoptosis, angiogenesis, and immune response [9]. Recent studies have described the role of miRNA expression in the initiation and progression of CRC and its response to different therapeutic strategies [10]. Thus, miRNAs can also be used as prognostic and predictive factors in the clinical setting of CRC in adjunct to the use of TNM stage, tumor grade, and tumor classification.

Here, we review the literature on inflammatory markers and miRNAs with an established role on survival rates, response to systemic chemotherapy, and other clinic-pathological parameters in CRC patients.

## 2. Literature Review

### 2.1. Inflammatory Pathway

**Figure 1:**
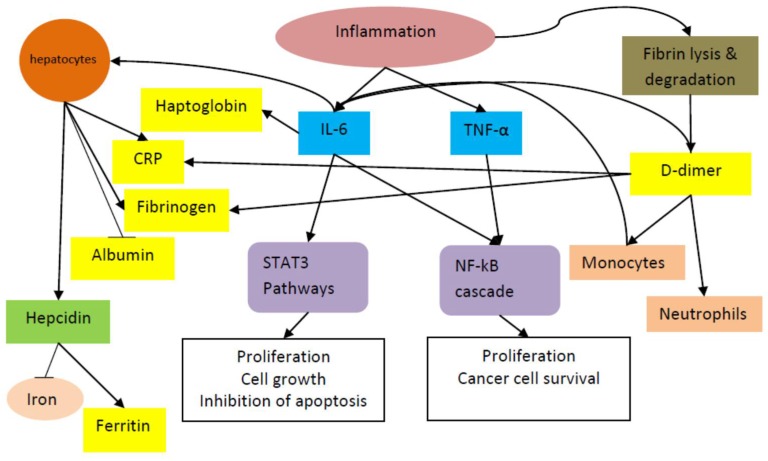
Inflammatory pathway involving inflammatory cytokines (shown in blue boxes) and acute-phase reactants (shown in yellow boxes). Only albumin is a negative acute-phase reactant, whose levels are reduced during inflammation (indicated by a bar instead of an arrow). STAT3: signal transducer and activator transcription 3; NF-κβ: nuclear factor κβ; TNF-α: tumor necrosis factor-α; IL-6: interleukin-6; CRP: c-reactive protein.

Cancer represents a state of high physiological stress, with tumor hypoxia/necrosis and local tissue damage. In order to counteract these changes, the body responds with a systemic release of proinflammatory cytokines and growth factors [11]. Figure 1 summarizes the inflammatory processes discussed below. Several proinflammatory cytokines have been shown to regulate cancer cell growth and thereby contribute to tumor promotion and progression. Interleukin-6 (IL-6) and tumor necrosis factor-α (TNF-α) are two cytokines which induce and maintain a systemic, low-grade inflammatory state [12,13]. IL-6 is primarily produced by monocytes and macrophages during acute inflammation and by T cells during chronic inflammation [14]. IL-6 binds to its receptor IL-6R and activates the signal transducer and activator transcription 3 (STAT3) pathway. A homodimer of phosphorylated STAT3 then translocates to the nucleus and induces the transcription of several target genes, promoting proliferation, cell growth, and the inhibition of apoptosis [15]. IL-6 and TNF-α also trigger the nuclear factor κB (NF-κB) cascade, thereby promoting the proliferation of cancer cells and rescuing the cancer cells from cell death [16]. Moreover, both signaling pathways increase enterocyte-specific nuclear localization of β-catenin, which contributes to colorectal cancer carcinogenesis [17].

CRP, an acute-phase plasma protein, is generated from hepatocytes in response to inflammatory cytokines, such as IL-1, TNF-α, and, in particular, IL-6 [18,19]. As part of the acute-phase response due to mild chronic inflammation associated with cancer, there is also a reduction in synthesis and an increase in degradation of albumin that results in hypoalbuminemia, whereas acute reduction in albumin concentrations in severe inflammations, such as burn and surgical trauma, is attributable to redistribution of albumin particles [20]. The end result is the nutritional and functional decline of patients, specifically those with advanced cancer. Haptoglobin is an acute-phase glycoprotein which is also produced in response to IL-6 [21]. Significantly elevated serum haptoglobin levels are seen in several types of cancers, including lung [22], breast [23], ovarian [24], and oral squamous cell cancers [25]. Haptoglobin contributes to increased oxidative stress and low-grade chronic inflammation [26].

D-dimer, a fibrin cleavage product, is a clinical marker that is used to diagnose pulmonary embolism [2]. D-dimer has been shown to have diverse effects on inflammatory processes and acute-phase responses, including neutrophil and monocyte activation; secretion of cytokines, including IL-6 and IL-1; and hepatic synthesis of acute-phase proteins, including fibrinogen and CRP [27,28]. Fibrinogen may enhance tumor cell proliferation, migration, and signaling through interaction with multiple integrin and non-integrin receptors and may also promote tumor angiogenesis by cooperation with growth factors, such as vascular endothelial growth factor and fibroblast growth factors [29].

In addition to its effect on acute-phase proteins, IL-6 induces the production of hepcidin by hepatocytes. Hepcidin in turn blocks iron release from macrophages, increases ferritin levels, and lowers transferrin saturation levels [30]. Ferritin, measured in routine blood examinations, is the primary iron-binding protein that exists both intracellularly and extracellularly [31]. Evidence has indicated that ferritin may play a role in cancer proliferation and immunosuppression, as well as therapeutic resistance [31].

#### 2.1.1. Acute-Phase Reactants

Multiple studies have evaluated the role of acute-phase reactants, including albumin, CRP, ferritin, fibrinogen, haptoglobin, and D-dimer, on prognosis in both early and advanced CRCs. Table 1 summarizes the studies that were conducted on these markers in CRC. The role of pretreatment serum albumin as a prognostic tool was demonstrated by two studies. The first concluded that baseline serum albumin inversely correlated with tumor-node-metastasis (TNM) stages [32], and the second demonstrated that serum albumin levels may be used to linearly predict the postoperative morbidity and mortality among resectable CRCs [33]. CRP was studied extensively in the setting of CRC. Studies have highlighted the potential impact of elevated CRP levels on worse progression free survival (PFS) [34] and overall survival (OS) [35,36] in metastatic CRC (mCRC) patients on palliative chemotherapy and best supportive care [37]. CRP was also found to be a strong prognostic factor of survival following resection of colorectal liver metastases [38]. Moreover, in CRC patients undergoing surgical resection, higher CRP levels correlated with worse disease free survival (DFS) [39], increased anastomotic leak, and increased mortality [40]. An elevated CRP-to-albumin ratio was also predictive of worse survival in mCRC patients [41] and CRC surgery patients [42]. These results suggest that CRP levels should be taken into consideration when selecting treatment regimens for patients with CRC.

Two studies evaluated the prognostic impact of ferritin in CRC. Both studies showed that high ferritin levels were associated with poor survival in both resectable CRC [31] and mCRCs [43]. It is worth noting that when stratified by TNM stages, ferritin levels remained statistically significant with only stage III patients [31]. Haptoglobin, on the other hand, was also associated with poor survival. Moreover, high serum haptoglobin levels were significantly increased in the CRC-distant metastasis (CRC-M1) group compared to the CRC-no metastasis (CRC-M0) group. The study further demonstrated that, combined with serum carbohydrate antigen 19-9 (CA19-9) levels and serum carcinoembryonic antigen (CEA) levels, serum haptoglobin levels accurately predicted CRC liver metastasis [6]. Also, high pretreatment D-dimer levels were shown to predict poor survival in CRC [44]. D-dimer levels were significantly increased in poorly differentiated tumors, specifically in more poorly differentiated tumors with a higher T stage [45]. Data on preoperative fibrinogen, however, was not conclusive. One retrospective study correlated preoperative fibrinogen with cancer severity indicators, such as presence of systemic metastasis (*p* < 0.001), depth of tumor invasion pT (*p* < 0.001), node involvement pN (*p* = 0.001), and CEA serum level (*p* < 0.001), but it did not predict patient prognosis after colorectal cancer surgery [46]. Another study revealed that elevated preoperative plasma fibrinogen levels correlated significantly with venous invasion, advanced stage, and postoperative distant metastases, but not with lymph node involvement [47]. The study also showed an independent association between elevated preoperative plasma fibrinogen levels and impaired OS in patients with colorectal cancer in a multivariate survival analysis [47].

#### 2.1.2. Inflammatory Cytokines

Interleukins are a type of cytokine that control growth and differentiation, cell migration, and inflammatory and anti-inflammatory responses by the immune system [49]. Several proinflammatory interleukins have been implicated in CRC. In patients undergoing surgery, IL-6 levels were higher in stage II CRC patients than in stage III patients. IL-6 levels were found to be positively correlated with CRP and CEA levels, as well as with pT4 stage disease. Also, IL-6 was significantly associated with OS in all stages of CRC, but DFS was significantly different only in the stage II disease group [50]. Pretreatment serum concentrations of IL-1β and IL-6, as well as TNF-α, predicted PFS regardless of CRP levels. Measurement of IL-1β and IL-6 may help identify early cancer progression among patients with CRP < 5 mg/L in routine practice [51]. IL-37, which is highly abundant in healthy tissue adjacent to malignant tissue, suppressed cell migration, invasion, proliferation, colony formation, and cancer stem cells through suppressing β-catenin. Thus, the lower the expression of IL-37, the more highly malignant the CRC seems to be. In fact, IL-37 levels were found to be independent prognostic factors of OS and PFS in CRC patients [52]. High IL-23 expression was associated with advanced pathological T stage (*p* < 0.001) and late TNM stage (*p* < 0.003). Elevated serum IL-23 levels were also associated with poor 5-year PFS and OS (*p* < 0.048 and *p* < 0.028, respectively). The study showed that high IL-23 expression was predominant during advanced invasion and late-stage CRC [53]. IL-17 levels, on the other hand, were not predictive of unfavorable clinical outcomes, but they correlated with CD8 and CD16 cell invasion, which, by themselves, are predictors of unfavorable outcomes [54].

TNF-α is known to activate NF-kβ to protect tumor cells, and its upregulation leads to resistance to apoptosis and induces drug resistance in certain cancers [55]. Nevertheless, their expression and prognostic role in CRC remains unclear [56]. In CRC patients who had undergone surgery for their primary cancer, the higher levels of TNF-α were specifically associated with CRC-specific mortality [57]. Also, TNF-α expression in surgically resectable CRCs was associated with differentiation (*p* = 0.019), TNM stage (*p* = 0.039), lymph nodes metastasis (*p* = 0.024), and lymph vascular invasion (*p* = 0.027). TNF-α levels were also associated with decreased DFS (*p* = 0.0209) and OS (*p* = 0.0163) [56]. Another study reported that cytokine levels and the CRP level are clinically relevant for CRC progression, and that measurement of TNF-α serum levels may help identify early cancer progression among patients with CRC [51].

#### 2.1.3. Blood Cell Ratios

Several studies questioned the role of various blood cell ratios, such as neutrophil to lymphocyte ratio (NLR), lymphocyte to monocyte ratio (LMR), and platelet to lymphocyte ratio (PLR), in predicting the clinical outcomes of colorectal cancer.

NLR denotes the balance between the inflammatory response and the antitumor immune function. Tang et al. investigated the association of NLR and survival outcomes in colorectal cancer patients with liver metastasis. Their results demonstrated that elevated pretreatment NLR was significantly related to poor OS (HR 2.17, 95% CI 1.82–2.58) and recurrence-free survival (HR 1.96, 95% CI 1.64–2.35) [8]. Haram et al. had the same results with respect to OS, as their study results proved that high NLR is a negative predictive factor, not only in mCRC tumors, but also in locally advanced tumors [4]. Kim et al. also showed that high NLR (≥3.0) is an independent risk factor predicting poor long-term outcomes in patients with stage III and IV, but not in stage I and II [58].

The prognostic significance of LMR was studied in multiple settings. In chemo-naïve mCRC, Lin et al. showed, using a sample size of 488 patients, that patients with high pre-chemotherapy LMR (≥3.11) experienced significant improvement in PFS (9.2 vs. 7.6 months, *p* < 0.001) and OS (19.4 vs. 16.6 months, *p* < 0.001) compared with patients with low pre-chemotherapy LMR. These results were also seen in the setting of resectable CRC, as a recent study published in March 2017 showed an independence of LMR in being a predictive factor of survival [59]. The study had a huge sample size of 1623 with a cut-off value of LMR 2.38. They were able to show that low LMR (<2.38) was associated with higher stage and grade of tumor, and more present in right-sided tumors. On the other hand, high LMR (≥2.38) was present in early stages and grades of CRC, left-sided tumors and patients had a better OS (*p* < 0.001) independent of age, TNM stage, and grade [3]. The predictive value of LMR was also questioned in CRC cases metastatic to the liver and treated with radiofrequency ablation [7]. Median OS was 55 months in patients with LMR > 3.96 and 34 months in patients with LMR ≤ 3.96 (*p* = 0.007). Time to recurrence (TTR) of metastatic lesions was 35 months in the group with LMR > 3.96 and 25 months in the other. Therefore, the study managed to support LMR being a novel predictor of outcomes in CRC [7]. 

A systematic review done by Tan et al. [60] included 15 studies and a total of 3991 CRC patients. The study analyzed the relationship between PLR and OS and DFS. The meta-analysis showed that elevated PLR was significantly associated with lower OS (pooled HR, 1.53; 95% CI, 1.24–1.89; *p* ≤ 0.001), DFS (pooled HR, 1.68; 95% CI, 1.07–2.62; *p* = 0.023), poor tumor differentiation (odds ratio (OR) 2.12; 95% CI, 1.45–3.08, *p* < 0.001)), the propensity toward depth of infiltration (OR 1.69; 95% CI, 1.20–2.39, *p* = 0.003), and recurrence (HR, 2.71; 95% CI, 1.31–5.60, *p* = 0.005). Therefore, the authors suggested that high peripheral blood PLR can be used as a predictor of OS and certain clinic pathological parameters in patients with CRC [60].

### 2.2. MiRNAs

During the recent years, it has become clear that aberrant miRNA expression has a functional role in CRC [10]. MiRNAs are evolutionarily conserved, single stranded, noncoding RNA molecules that bind target miRNAs and prevent their translation. Specific miRNAs can act as either tumor suppressors or oncogenes, depending on the cellular environment in which they are expressed [10]. MiRNAs have been found to be dysregulated in various types of cancers, including CRC, and their functions have been linked to many of the processes involved in tumorigenesis, from initiation to progression and metastasis [10]. Interestingly, a number of studies have described a correlation between the expression pattern of miRNAs and the diagnosis, prognosis [61], and therapeutic outcome in CRC [62,63,64,65,66,67]. These data suggest that miRNAs may be potential molecular classifiers, early detection biomarkers, and therapeutic targets for CRC [67]. An extraordinary advantage of miRNAs is represented by their presence in stable form in body fluids, including blood, making them promising noninvasive biomarkers. Accumulating data have indeed revealed that circulating miRNAs can serve as diagnostic, prognostic, and predictive biomarkers for CRC. In this review, we mainly focus on examples of miRNAs with potential prognostic and predictive value (summarized in Table 2).

Chen et al. revealed that high levels of serum miR-155 is associated with resistance to adjuvant chemotherapy mFOLFOX (5-fluorouracil, leucovorin, and oxaliplatin) [68]. Furthermore, elevated levels of circulating miR-155, together with miR-200c and miR-210, implicate local recurrence and distant metastasis [68]. Aberrant expression of serum miR-19a was shown to predict resistance to first line FOLFOX chemotherapy in advanced CRC patients. These results were concordant with a recent study showing that serum exosomal miR-19a was predictive of poor prognosis [64,65]. In addition to chemotherapy resistance, a study published in 2017 showed that postoperative plasma miR-31, miR-141, and miR-16 can be used as potential biomarkers for early prediction of disease recurrence following resection [61]. A recent meta-analysis published in 2018 also confirmed the prognostic value of circulating miR-141 in CRC [69]. Moreover, high expression of miR-126 was correlated with bevacizumab resistance in mCRC [66] and that of miR-345, with lack of response to third line cetuximab and irinotecan [67]. 

We have highlighted thus far in this review the role of inflammatory biomarkers and miRNAs in CRC prognosis. It is thus important to note that inflammatory pathways in CRC could be regulated by miRNAs [70,71]. For example, a modulatory role of miR-105 in TNF-α-induced CRC metastasis was described by Shen et al. [72]. Moreover, miR-34a was shown to be a crucial regulator of IL6/STAT3 signaling in CRC [73].

In summary, the emerging role of miRNA as promising non-invasive prognostic and predictive biomarkers in CRC is paramount. However, despite this promise and because of the inconsistency and little reproducibility among the published studies assessing the role of miRNAs as biomarkers, their clinical use is still in early stages of development and, accordingly, more optimized research is still needed to strengthen their clinical value.

## 3. Materials and Methods

Retrieval of studies was performed through Medline on PubMed by using the following “meSH terms”: Colorectal neoplasm, Inflammatory biomarkers, C-reactive protein, Albumin, Serum Ferritin, Fibrinogen, D-dimer, Haptoglobin, Tumor necrotic factor, and Interleukins. We also used “key words”, as well as manual search across abstracts obtained for proper selection criteria. The key words mainly assisted in retrieving articles addressing the prognostic and predictive value of blood count ratios and miRNA in CRC.

- Selection criteria consisted of the following:
- English published papers
- Published between 2008–2018
- Published in peer reviewed journals
- Papers assessing prognostic role of inflammatory markers and predictive miRNAs in different settings of colorectal cancer

## 4. Conclusions

**Figure 2:**
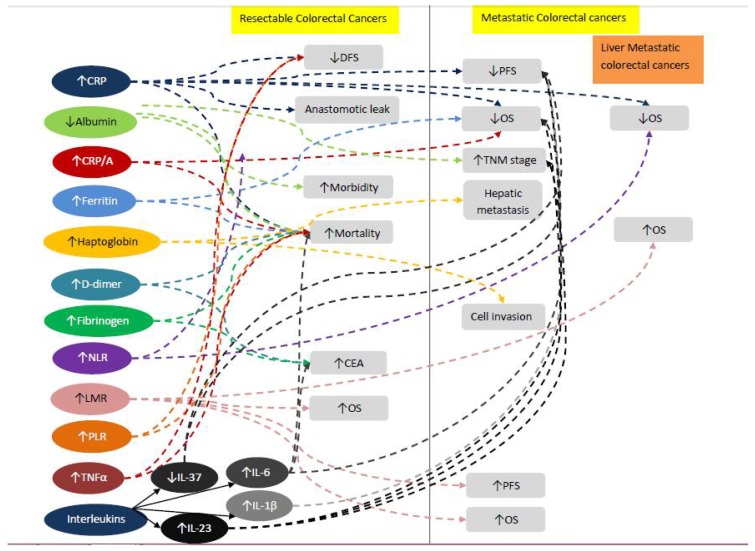
Current evidence correlating inflammatory biomarkers to survival and outcomes in resectable and metastatic colorectal cancers. NLR: neutrophil to lymphocyte ratio; LMR: lymphocyte to monocyte ratio; PLR: platelet to Lymphocyte ratio.

Even though prognosis of CRC patients is currently predicted by surgical resection and pathological analysis of specimens, these methods are considered suboptimal. A growing body of literature has investigated laboratory markers as prognostic factors adjunct to pathological staging. Data from literature prove the hypothesis that systemic inflammation is the maestro of malignant cell evolution and tumor advancement. The change in the level of acute-phase reactants, such as CRP, ferritin, fibrinogen, D-dimer, haptoglobin, and albumin, was found to be associated with a dismal prognosis and survival outcome in colorectal cancer patients in various settings of the disease (summarized in Figure 2). Little is reported about the clinical use of these inflammatory biomarkers for CRC patients, so it would be interesting to study their sensitivity and specificity alone and combined in different cohorts. MicroRNA expression also showed a substantial correlation with the tumor biology and behavior, and mounting evidences suggest that aberrant miRNA expression can play a crucial role in CRC and can serve as potentially useful biomarkers for predicting prognosis and clinical response and accordingly stratify CRC patients for optimal drug selection. On the other hand, miRNAs have not been yet clinically utilized due to the inconsistency and poor reproducibility upon their detection in circulation. In order to overcome these challenges, an optimal strategy for miRNA detection, which focuses on the variability in the patients’ characteristics, experimental design, as well as the isolation and detection methodologies, could be followed, as discussed in Nassr et al. [74]. As such, large-scale prospective studies are needed to validate these potential prognostic and predictive biomarkers, as well as to determine their levels, sensitivity, and specificity in CRC patients with different primary tumor locations.

## Figures and Tables

**Table 1:** Studies evaluating the prognostic impact of acute phase proteins in colorectal cancer (CRC).

Study	Study Design	Population	Aim	Results
Albumin				
Gonzalez-Trejo et al. [32]	Retrospective	1464 CRC patients	To define the prognostic role of baseline serum albumin in CRCs across tumor-node-metastasis (TNM) stages	Baseline serum albumin was inversely correlated with TNM stages.
Chiang et al. [33]	Retrospective	3732 CRC surgery patients	To evaluate according to albumin level the postoperative morbidity and mortality in CRC patients	Morbidity decreased by 7.3% and mortality by 15.6% with every 0.1 g/dL increase in albumin level
CRP				
Mik et al. [40]	Retrospective	724 CRC surgery patients	Development of anastomotic leak and OS	Anastomotic leak: 4.6% CRP had sensitivity of 75% and a specificity of 91% in determining anastomotic leak. CRP levels were also found to be significantly higher in patients who died in the postoperative phase
Riedl et al. [37]	Retrospective	258 mCRC patients undergoing palliative chemotherapy or immunotherapy	6-month PFS and overall response rate (ORR) during first, second, and third line treatment, and 6-month OS during best supportive care (BSC)	Higher CRP levels predicted worse PFS in the first chemotherapy lines and in BSC (hazard ratio (HR) = 1.49 (*p* < 0.0001 first line); HR = 1.25 (*p* = 0.007 second line); HR = 1.09 (95% CI 0.81–1.48, *p* = 0.552 third line and HR = 1.43 (*p* = 0.002 in BSC))
Nagai [39]	Review of prospective database	1174 patients with stage I, II, or III CRC who underwent R0 resection	Identify the prognostic factors from preoperative routine blood data that have a significant relationship with DFS	A higher CRP level was significantly correlated with worse DFS upon univariate analysis but not upon multivariate analysis
Artac et al. [34]	Retrospective	90 mCRC patients receiving folinic acid, bolus/continuous fluorouracil, and irinotecan with bevacizumab (FOLFIRI-Bev)	Identify the efficacy of CRP on PFS in patients receiving FOLFIRI-Bev	At multivariate analysis, CRP was shown to be an independent prognostic factor. The median PFSs of the patients with normal and above the upper limit of normal were 11.3 versus 5.8 months, respectively (*p* = 0.022)
Thomsen et al. [35]	Review of prospectively collected data	393 mCRC patients from phase III trial of cetuximab with continuous or intermittent fluorouracil, leucovorin, and oxaliplatin (Nordic FLOX) versus FLOX alone (NORDIC-VII trial) receiving 1st line therapy	Identify the effect of CRP levels on PFS and OS	In the four categories of baseline serum CRP level (≤10, 11–30, 31–60, and >60 mg/L), median PFS was 8.9, 7.6, 8.2, and 6.6 months, respectively (log rank test, *p* < 0.001) and median OS was 24.3, 20.6, 17.1, and 12.3 months, respectively (log rank test, *p* < 0.001)
Shibutani et al. [41]	Retrospective	99 mCRC patients undergoing palliative chemotherapy	Evaluate the significance of the C-reactive protein to albumin (CRP/ALB) ratio in colorectal cancer	The OS rate was significantly worse in the high pretreatment CRP/ALB ratio group than in the low pretreatment CRP/ALB ratio group (*p* = 0.0009)
Kostner et al. [38]	Retrospective	492 CRC patients with liver metastases	Evaluate the prognostic role of CRP in colorectal cancer patients with liver metastasis	Preoperative CRP > 10 mg/L was a strong predictor of worse survival (HR = 1.72, 95% CI 1.84–2.50, *p* < 0.01). Patients with CRP ≤ 10 mg/L had a median survival of 4.27 years compared to only 47 days in patients with CRP ≥ 30 mg/L (*p* < 0.01).
Ishizuka et al. [42]	Retrospective	626 CRC patients who underwent elective surgery	Estimate the clinical significance of the CRP/ALB ratio for prediction of postoperative survival	Multivariate analysis showed that CRP/ALB ratio was associated with OS (hazard ratio 2.596; 95% confidence interval 1.603–4.204; *p* < 0.001). The study also showed a significant difference between patients with low CAR and those with high CAR in Kaplan–Meier analysis and log rank test (*p* < 0.001).
Casadei Gardini et al. [36]	Secondary analysis on patients enrolled in the phase III prospective multicenter randomized “Italian Trial in Advanced Colorectal Cancer (ITACa)”	132 CRC patients. Samples were collected at baseline and 2 months after starting 1st line chemotherapy	To assess high-sensitivity C-reactive protein (hs-CRP) levels at diagnosis and their impact on PFS and OS.	High levels of hs-CRP (≥13.1 mg/L) were associated with poorer median PFS (*p* < 0.0001) and OS (*p* < 0.0001) than low hs-CRP levels (<13.1 mg/L). hs-CRP values in 107 patients were evaluated again after 2 months of therapy, revealing that patients with low hs-CRP levels in both baseline and second serum samples had the best median PFS and OS.
Ferritin				
Lee et al. [43]	Retrospective	120 mCRC patients	To investigate the prognostic impact of serum ferritin on survival in patients with mCRC	High serum ferritin levels were associated with increased mortality after mCRC treatment, with increased hazard ratio and poor survival (ferritin ≥ 150 ng/mL; HR 1.763, 95% CI 1.169–2.660, *p* = 0.007)
Tingting et al. [31]	Prospective	514 CRC surgery patients	To validate the prognostic significance of preoperative serum iron metabolism parameters in non-metastatic colorectal cancer patients treated with curative resection.	High serum ferritin levels had a 2.21-fold increase in mortality compared with patients with the lowest quartile ferritin
Haptoglobin				
Sun et al. [6]	Retrospective	475 CRC patients and 152 healthy volunteers	To assess the potential of serum haptoglobin as a marker for early detection of CRC metastasis	The study showed that serum haptoglobin levels were 89.1% sensitive and 85.8% specific in detecting hepatic metastasis
Fibrinogen				
Pedrazzani et al. [46]	Retrospective	653 CRC surgery patients	To evaluate the clinical significance of the preoperative fibrinogen plasma level as a prognostic marker after surgery for colorectal cancer	OS and tumor-related survival were significantly higher in patients with fibrinogen values ≤ 400 mg/dL (*p* < 0.001). Elevated fibrinogen levels did not remain statistically significant for either overall (*p* = 0.313) or tumor-related survival (*p* = 0.355) upon multivariate analysis.
Tang et al. [47]	Retrospective	341 CRC surgery patients	To evaluate the association between preoperative plasma fibrinogen levels on clinicopathologic parameters and OS in patients after curative resection with colorectal cancer	Elevated plasma fibrinogen levels were associated with advanced tumor stage (*p* = 0.008), venous invasion (*p* = 0.006), and postoperative distant metastases (*p* < 0.001). Multivariate analysis showed that preoperative plasma fibrinogen level was prognostic for survival (*p* = 0.029)
D-dimer				
Lu et al. [44]	Meta-analysis of 15 studies	2283 CRC patients	To provide insight into the prognostic role of pretreatment D-dimer levels	High pretreatment plasma D-dimer predicts poor survival of CRC HR of 2.167 (95% CI: 1.672–2.809, *p* < 0.001)
Yu et al. [48]	Cross-sectional study	120 CRC patients	To look into the prognostic values of D-dimer levels in cancer patients	D-dimer levels are significantly higher in cancer patients compared to healthy controls. D-dimer levels correlated with poor prognosis and survival rate
Tekesin et al. [45]	Prospective study	165 CRC surgery patients	To relate the preoperative D-dimer and CEA levels of patients with CRC undergoing surgical resection to the prognosis and postoperative survival rate	Increased D-dimer and CEA levels were associated with significant decrease in postoperative survival rate and prognosis

**Table 2:** miRNAs that influence CRC prognosis.

miRNA	Predictive Role in CRC	Reference
miR-155	Resistance to adjuvant chemotherapy mFOLFOX	Chen et al. [68]
miR-155, miR-200c, and miR-210	Local recurrence and distant metastasis	Chen et al. [68]
miR-19a	Resistance to first line FOLFOX	Chen et al. and Matsumura et al. [64,65]
miR-31, miR-141, and miR-16	Early prediction of disease recurrence following resection	Yuan et al. [61]
miR-141	Prognosis	Gao et al. [69]
miR-126	Bevacizumab resistance	Hansen et al. [66]
miR-345	Insensitivity to third line cetuximab and irinotecan	Schou et al. [67]

## Abbreviations

CRC	Colorectal cancer
CRP	C-reactive protein
TNF-α	Tumor necrosis factor α
IL	Interleukin
TNM	Tumor-node-metastasis
NLR	Neutrophil to lymphocyte ratio
LMR	Lymphocyte to monocyte ratio
PLR	Platelet to lymphocyte ratio
M0	No metastasis
M1	Distant metastasis
FOLFOX	5-Fluorouracil, leucovorin, and oxaliplatin
CA19-9	Carbohydrate antigen 19-9
CEA	Carcinoembryonic antigen
OS	Overall survival
PFS	Progression free survival
DFS	Disease free survival
ORR	Overall response rate
HR	Hazard ratio
FOLFIRI	Folinic acid, bolus/continuous fluorouracil, and irinotecan
Bev	Bevacizumab
